# A New Method of Placing the Old-Fashioned Pivot Crown

**Published:** 1885-01

**Authors:** E. C. Redgell


ARTICLE X.

### A NEW METHOD OF PLACING THE OLD-FASHIONED PIVOT CROWN.

BY DR. E. C. REDGELL.

I have quite recently struck upon a plan for setting crowns, which for simplicity, strength, durability and neatness I don't think is excelled by any of the modern methods. I will thus describe it: Consider a root in a healthy state, file or grind it down some distance below the gum, burr or ream out the canal as near the apex of the root as possible, cut the walls around the orifice freely, if the wall of the root is no thicker than paper at the orifice it makes no difference; select a common pivot tooth of size and color to correspond with the natural teeth; measure the depth of the canal and socket in pivot tooth and cut from one of Howe's screw posts a pivot of the required length; dry and fill, about onefourth full of cement (I prefer Caulk's), and while it is yet quite soft press your pivot with the crown on it home; leaning the crown in any desired position at this time, as this will determine its future position; hold it so for a few minutes, then remove the crown, leaving the post fixed in the cement.

Now fill the remainder of the canal with gold or amalgam flush (I prefer amalgam), and while soft put the crown on the post and give it a few gentle taps; you can make a magnificent fit.

Then fill the socket in the crown half full of cement, put it on the pivot, and press home, holding it for a few minutes, when your work is done.

If you should anticipate future trouble and wish to treat the root through the canal, file the threads off the post that enters the crown before putting it on, then the crown may be very easily removed at any time by taking a pair of forceps and turning it, which will sever it from the cement; with a pair of pliers you can unscrew the post and have access to the root without irritating it or breaking any of your work.

By bending the post, grinding the crown or not, you can give most any of the divergencies necessary for the crown. By this method the root can be entirely covered with the filling; you can take weakest kind of roots and put strong and durable work on them.—*The Dental Advertiser.*

